# Benzodiazepine usage and patient preference for alternative therapies: A descriptive study

**DOI:** 10.1002/hsr2.116

**Published:** 2019-02-21

**Authors:** Fatema‐Tun‐Naher Sake, Keith Wong, Delwyn J. Bartlett, Bandana Saini

**Affiliations:** ^1^ The School of Pharmacy, Faculty of Medicine and Health University of Sydney Sydney Australia; ^2^ Faculty of Medicine and Health The University of Sydney Sydney Australia; ^3^ Woolcock Institute of Medical Research The University of Sydney Sydney Australia; ^4^ Department of Respiratory and Sleep Medicine Royal Prince Alfred Hospital Camperdown Australia

**Keywords:** behavioural therapies, beliefs, benzodiazepine, chronic use, patient factor, primary care, withdraw

## Abstract

**Background and aims:**

The prevalence of chronic benzodiazepine use in primary care settings remains high despite clear evidence of adverse health outcomes resulting from long‐term use and the availability of effective alternative behavioural therapies. Eliciting factors influencing past or current usage experience of benzodiazepine users and their future behavioural intention regarding discontinuation or alternative behavioural therapy adoption could be useful in developing informed strategies facilitating successful benzodiazepine withdrawal in long‐term users. The aim of this study was to identify patient factors influencing their current long‐term benzodiazepine use, past withdrawal attempt, and future intention to trial safer alternative behavioural therapies. Additionally, the study also aimed to explore patients' preference for information sources on behavioural therapies.

**Methods:**

Point of purchase surveys were conducted with patients obtaining benzodiazepines from selected pharmacies across New South Wales (NSW), Australia. Survey items included the Beliefs about Medicines Questionnaire (BMQ‐specific), questions about patient's sociodemographic characteristics, as well as their views about long‐term benzodiazepine use and behavioural therapies.

**Results:**

Seventy‐five patients were recruited from 12 pharmacies across New South Wales (NSW). The surveys were conducted from November 2016 to July 2017. The mean (±SD) age of the participants was 54.3 (±16.7) with a range of 23 to 86 years, and 67% of the participants had been using the benzodiazepine for at least 1 year. Lower‐education levels, stronger beliefs about the necessity of use, and lower concerns about ongoing benzodiazepine use were significantly associated with prolonged use. Sixty‐four percent of the participants were not interested in behavioural therapies, and there was a significant relationship between the participants' future preference for behavioural therapies and their concerns about the potential adverse effects of benzodiazepines. A majority of the participants rated general practitioners (GPs) as their first choice and pharmacists as the second choice for discussing behavioural therapies.

**Conclusions:**

Specific individual sociodemographic characteristics of benzodiazepine users and their medication‐related beliefs influence their current benzodiazepine usage and future intention to trial behavioural therapies as an alternative to their benzodiazepines. Based on the reported preferences of benzodiazepine users in this study, developing and evaluating GP‐pharmacist collaborative services to improve the uptake of behavioural therapies as an alternative to benzodiazepines can be recommended.

## 1 INTRODUCTION

Benzodiazepines are widely prescribed for their hypnotic,^1^ anxiolytic,^2^ muscle relaxant, and antiepileptic indications.^3^ Despite their common use, concerns remain over their long‐term safety. Pharmacoepidemiological data indicate that prolonged use of benzodiazepines is associated with an increased risk of falling,^4^, ^5^ therapeutic dose dependence,^6^ and an increased incidence of dementia.^7^ Benzodiazepine exposure is also associated with an increased risk of physical disability.^8^ Recent cohort studies suggest an increased risk of exacerbations in benzodiazepine users with asthma and a higher likelihood of episodes of pneumonia and related mortality.^9^, ^10^ A recent systemic review also highlighted an overall increased risk of all‐cause mortality in benzodiazepine users.^11^ Inappropriate use of benzodiazepines in Australia has been recently reported to lead to high costs of managing adverse events in residential aged care facilities.^12^

In light of the well‐established side effect profile of benzodiazepines, prescribing guidelines do not recommend their prolonged use. The Royal Australian College of General Practitioners (RACGP) recommends that benzodiazepines should not be prescribed for longer than 4 weeks.^13^ For patients requiring ongoing treatment, behavioural therapies such as cognitive behavioural therapy offer a promising alternative to the benzodiazepine for several conditions. For example, mounting evidence suggests that behavioural treatments produce comparable efficacy with benzodiazepines and have a longer‐lasting effect in patients with insomnia.^14^, ^15^, ^16^ Similarly, research evidence highlights better or equivalent efficacy profiles for behavioural therapies over benzodiazepines for managing anxiety disorder, panic disorder, and dysthymia.^17^

The benefits of de‐prescribing benzodiazepines have been highlighted in several studies. For example, in elderly nursing home residents, benzodiazepine withdrawal significantly improved memory and cognitive functioning compared with those who continued to take benzodiazepines; withdrawal did not give rise to anxiety, agitation, or sleeplessness.^18^ Health care utilization and hospital admission costs resulting from traffic accidents and falls attributable to benzodiazepine use can be reduced through successful discontinuation^19^; thus, benzodiazepine discontinuation can also have an economic benefit.

Despite the prescribing guidelines, the listed adverse effects of prolonged benzodiazepine use and robust evidence supporting behavioural therapies over benzodiazepines, the long‐term use of benzodiazepines remains high. While the pattern of benzodiazepine prescribing varies globally, prolonged use of benzodiazepines is a common phenomenon in the United Kingdom, Europe, and North America.^20^ A US study reported that in 2008, approximately 5.2% of US adults (18‐80 years) had used benzodiazepines.^21^ Reports comparing data on benzodiazepine use from the US National Health and Nutrition Examination Survey (NHANES) between 1999 and 2014 highlight significantly increased use (2.0% of respondents in 1999‐2000 to 4.2% in 2013‐2014), mostly driven by medium to long‐term users.^22^ Although there has been a modest decline in the volume of benzodiazepine prescriptions dispensed annually, overall, there is still a high level of long‐term benzodiazepine use in Australia.^23^, ^24^, ^25^ For example, in the case of insomnia, almost 80% of patient presentations result in a prescription for benzodiazepine derivatives (such as temazepam, oxazepam, diazepam, and nitrazepam), and this figure has remained stable over the past 10 years.^26^, ^27^

Both patient‐ and prescriber‐related factors appear to be linked with prolonged benzodiazepine use. Prescribers' perceptions and attitudes have been reported to be a key factor leading to prolonged benzodiazepine use.^28^ Recently, an Australian study highlighted that physicians often believe that patients taking benzodiazepines are unlikely to be willing to withdraw their medication and, thus, renew prescriptions without offering discontinuation or withdrawal plans.^29^ Individual patient factors can also affect the length of benzodiazepine use; data indicate that being older, lonely, less educated, as well as having a poorer mental health profile and lower perceived general health status are associated with prolonged benzodiazepine use.^30^, ^31^

Other patient‐related factors that can influence ongoing use of benzodiazepines include beliefs and attitudes of the users towards their medication.^32^ This has been proposed based on the well‐known Health Belief Model (HBM), which suggests that patients' beliefs about their health issues, perceived benefits of and barriers to action, and self‐efficacy explain engagement in health‐promoting behaviours while the actual action is triggered by cues.^33^ In fact, instruments such as the Beliefs about Medicines Questionnaire‐specific version (BMQ‐specific) have been designed and validated to assess patients' beliefs and attitudes about their medications.^34^ The BMQ‐specific consists of two 5‐item scales themed as (1) *necessity* and (2) *concerns*. While the necessity theme assesses individuals' beliefs about the necessity of using their medication, the concerns theme evaluates their concerns about the medication (eg, side effects, fear of dependence, and among others). Thus, in the case of benzodiazepine use, it may be hypothesised that the balance between the concerns about the risk related to continued use of benzodiazepines versus the beliefs about the necessity of the medication may dictate a patients' past, current, and future decisions related to their medications, eg, attempting withdrawal, seeking safer alternatives, or continuing use. Gauging the patients' position on these opposing belief sets (necessity vs concerns) can allow health professionals to employ targeted information provision or counselling to enhance effective withdrawal. Where medication discontinuation is desirable, based on the reasoning behind the BMQ‐specific, beliefs that foster medication discontinuation (higher‐risk perception about the medication and a lesser perceived necessity for use) could serve as a triggering point at which patients can be empowered and pragmatic strategies offered to facilitate withdrawal. In support of this notion, a qualitative study in patients with anxiety indicated that strong beliefs around the necessity of using benzodiazepines and a lower level of concern about long‐term use were associated with resistance to benzodiazepine discontinuation.^35^ Similarly, another qualitative study reported that individuals with higher concerns about the risk of taking benzodiazepines were more likely to attempt ceasing the medication of their own volition and were often more interested in trying behavioural interventions.^36^ Exploratory work mapping such beliefs about benzodiazepines and intention to continue use is clearly required for successful discontinuation trials.

Although there has been considerable research to characterize such individual sociodemographic and belief factors, there are, to date, few real‐life studies available on patients' perceptions around benzodiazepine use and withdrawal. Minimal attention has been paid to the complexity of factors affecting the decision making involved in withdrawing benzodiazepines. Successful and sustained withdrawal behaviours require a prior intention, and past behaviour may contribute to this future intention.^37^ In most research reporting trials of benzodiazepine weaning off, the patients' willingness to withdraw benzodiazepines and intention or readiness to adopt alternative therapies are assumed but not explored.^38^, ^39^, ^40^ It is not surprising that in many of these reported trials, benzodiazepine discontinuation rates remain less than 65%, with low‐response and high‐dropout rates.^38^, ^39^, ^40^ Patients' willingness to stop the medication and try behavioural therapies might be a key factor that affects successful discontinuation.

Therefore, the present study primarily aimed to investigate the association of participants' sociodemographic variables and beliefs about the benzodiazepine, with their benzodiazepine use behaviours, including ***past*** (previous benzodiazepine withdrawal attempts), ***current*** (long‐term benzodiazepine use, ie, using benzodiazepines for at least 1 year), as well as ***future*** behaviours (willingness to trial behavioural substitutes in the future). Additionally, the study aimed to explore the patients' source preference for information about alternative behavioural therapies. The findings of this study will potentially inform effective, patient‐centred, and evidence‐based interventions for reducing long‐term benzodiazepine use and disseminating behavioural therapies with greater scope and sustainability.

## 2 METHODS

### 2.1 Study design

**Figure 1:**
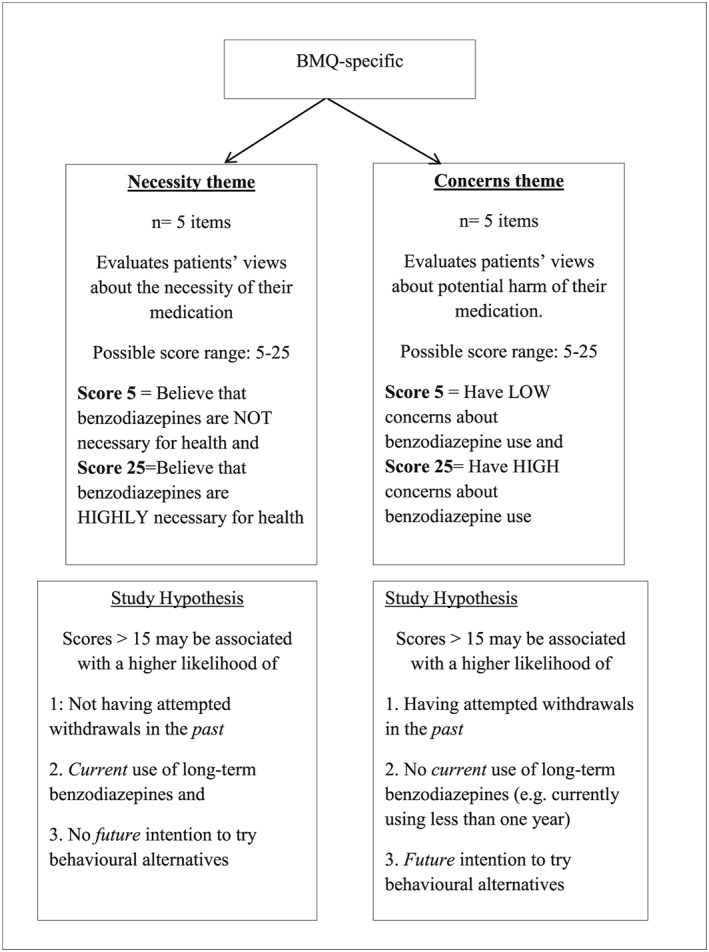
Beliefs about Medicines Questionnaire‐specific (BMQ‐specific)^34^ questionnaire items and scoring

**Figure 2:**
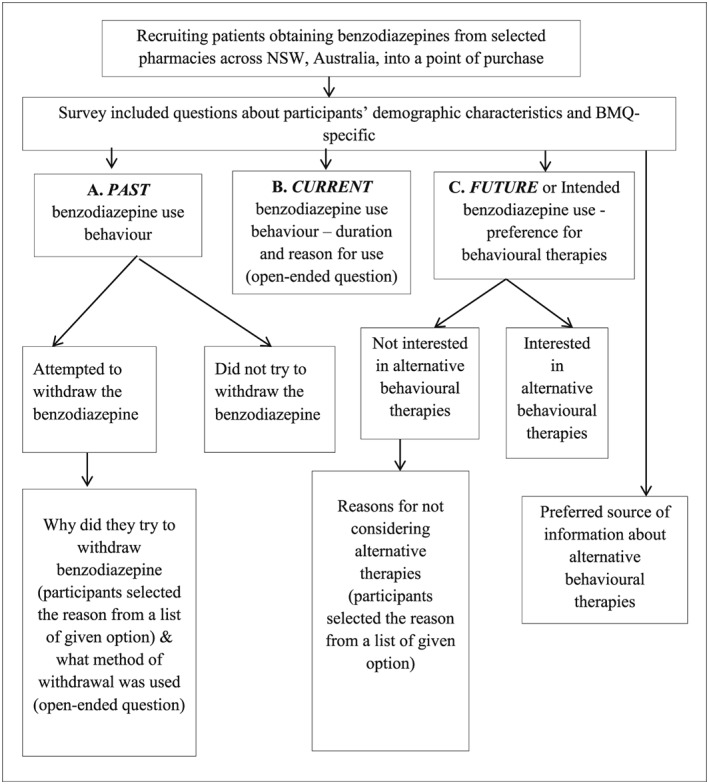
Diagrammatic representation and flow of questions

Given that this study aimed to explore real‐life behaviour, a point of purchase survey of benzodiazepine consumers was used. This method allows researchers to study consumer behaviour for informing interventions and policy development.^41^, ^42^ The survey questionnaire was developed by reviewing relevant literature,^35^, ^43^, ^44^ and the survey items included questions related to participants' demographic characteristics as well as their perspectives about the chronic use of benzodiazepines and alternative therapies. The developed questionnaire was reviewed by psychology, pharmacy practice, and sleep researchers for relevance and wording of the questionnaire items. A validated 10‐item BMQ‐specific was also used to assess participants' personal views about their benzodiazepines (Figure 1).^34^ Figure 2 outlines the diagrammatic representation and flow of questions used in the survey. This study was approved by The University of Sydney Human Research Ethics Committee (HREC: 2014/1020).

### 2.2 Participants

Pharmacies throughout the Australian state of New South Wales (NSW) were invited to participate utilizing a convenience sampling approach (ie, pharmacies known to the research team). The surveys were conducted from November 2016 to July 2017. Consent for a researcher (FTNS) to be present in a pharmacy was obtained from pharmacy owners. Potential participants included adult (18 years old and above) patients who were supplied benzodiazepines upon prescription on the day of the survey. The Anatomical Therapeutic Chemical (ATC) Classification System^45^ was used for the classification of drugs of interest, ie, N03AE (antiepileptics, benzodiazepine derivatives), N05BA (anxiolytics, benzodiazepine derivatives), and N05CD (hypnotics, benzodiazepine derivatives). N05CF (benzodiazepine‐related drugs, Z‐drugs) were not included in this study. After providing informed consent, participants filled out the survey questionnaire (participants had the option to ask questions to the pharmacist or research team member for unclear items), placed the completed survey in a sealable prestamped envelope, and handed this over to the research team member (FTNS) or mailed the completed survey back to the researchers. Participants were given the option to complete the survey questionnaire either at the pharmacy or at home. The time that it would take participants to complete the survey was tested by the researchers and estimated to be between 15 and 20 minutes.

### 2.3 Analysis

Completed survey questionnaires were assigned a code before initiating the analysis. Data were entered into the IBM SPSS Statistics package for Windows, Version 22.0 (Released 2013, Armonk, New York: IBM Corp), and entered data were checked for normality using the Kolmogorov‐Smirnov (K‐S) test^46^ as well as through visual inspection of histograms.^47^ Descriptive analyses were conducted to examine the demographic characteristics of participants. Correlation coefficients (Spearman's correlation coefficient or Phi coefficient for two binary variables) were used to describe (1) associations between current long‐term benzodiazepine use (using benzodiazepines for at least 1 year) and the following variables: age, sex, level of education, benzodiazepines being used, length of action of the benzodiazepine, and reason for taking the benzodiazepine; (2) associations between the BMQ score (concerns and necessity themes) and long‐term benzodiazepine use (current behaviours), benzodiazepine withdrawal attempt (past behaviours), and preference for behavioural therapies (future behaviours); and (3) associations between past, current, and future intended behaviours. Finally, binary logistic regression was conducted to determine whether past benzodiazepine withdrawal attempts and future preference for behavioural therapies differed with demographic or beliefs‐related variables that were significant in the correlation analysis or previous literature.^48^, ^49^, ^50^, ^51^ All tests were two‐sided with a significance level of 0.05.

## 3 RESULTS

The results are outlined with reference to our study's stated aims; these included exploring the association of participant's benzodiazepine usage behaviours with their sociodemographic characteristics and medication‐related beliefs. A secondary aim of the study was to confirm participants' preferences for information sources on alternative behavioural techniques. Accordingly, in the results section, firstly participants' demographics and then their scores on the BMQ‐specific questionnaire^34^ are presented. This is followed by presenting associations between the sociodemographics and BMQ scores with participants' benzodiazepine usage behaviours, which are presented in sequence: ***past** (previous withdrawal attempt)*, ***current** (long‐term use of benzodiazepine),* and ***future*** *(intention to try behavioural alternatives)* behaviours. Finally, participants' preferred sources of information for behavioural therapies are descriptively reported.

A total of 75 participants (67% female) out of 107 approached (70% response rate) were recruited for this study from 12 different pharmacies across NSW. Of these 12 pharmacies, 10 were medium‐sized (150‐350 m^2^ floor space) and two were large‐sized (350‐500 m^2^ floor space) pharmacies; eight were independently owned; 10 were from metropolitan, and two were from regional areas. All the participants completed the survey questionnaire at the pharmacy, even though the option to complete at home and mail back was available. The mean (±SD) age of the participants was 54.3 (±16.7), with a range of 23 to 86 years. Seventy‐one percent of the participants had at least high school level education. Sixty‐seven percent of the participants had been taking benzodiazepines for at least 1 year. The reported reasons for benzodiazepine use were sleep disorders (59%), psychiatric disorders (31%), pain management (7%), and other problems (4%). For the BMQ scale, the mean score (±SD) calculated for the necessity theme was 17.7 (±5.6), and the concerns theme was 13.6 (±4.7). While 65% of the sample had strong beliefs in the necessity of their benzodiazepine use as indicated by a BMQ‐specific score of greater than 15, only 40% reported strong concerns about the potential adverse effects of the medication (BMQ‐specific scores greater than 15). Descriptive statistics for the participants are shown in Table 1.

**Table 1:** Descriptive statistics for demographic characteristics of the participants (*n* = 75)

Demographic Variables	Number (%)
Females	50 (67)
Level of education	
No education	1 (1)
Primary school education	21 (28)
High school education	23 (31)
Vocational education	11 (15)
Tertiary education (graduate or postgraduate level)	19 (25)
Benzodiazepines purchased on the day of the survey	
Diazepam	43 (57)
Temazepam	28 (37)
Oxazepam	4 (5)
Using benzodiazepines for at least 1 year	50 (67)
Reason for benzodiazepine use	
Using benzodiazepine for sleep disorders	44 (59)
Using benzodiazepine for psychiatric disorders	23 (31)
Using benzodiazepine for pain	5 (7)
Using benzodiazepine for other problems	3 (4)

### 3.1 *Past* benzodiazepine use behaviours (withdrawal attempts)

In the sample, 47% (*n* = 35) of the participants had attempted to withdraw the benzodiazepine, and in these cases, sudden cessation of the benzodiazepine was the most commonly reported strategy applied to withdraw the medication. The reported reasons for attempting benzodiazepine withdrawal included concerns about health (49%), fear of developing dependence (40%), doctors' suggestion (37%), and other reasons (17%). Details about the reasons and strategies for withdrawing the benzodiazepine are presented in Table 2.

**Table 2:** Reasons and strategies for attempting benzodiazepine withdrawal (*n* = 35)

Reasons and Strategies for Attempting to Discontinue Benzodiazepines	Number (%)
Reasons for attempted benzodiazepine withdrawal	
Concerns about health	17 (49)
Fear of developing dependence	14 (40)
Doctors' suggestion	13 (37)
Other reason	6 (17)
Strategies applied to withdraw benzodiazepines	
Stopping the benzodiazepine	15 (20)
Reducing the dose	5 (7)
Exercise	3 (4)
Taking alcohol	2 (3)
Trying sleep hygiene	2 (3)
Using a herbal product	2 (3)
Changing lifestyle	2 (3)
Trying an antidepressant	1 (1)
Trying behavioural strategies	1 (1)
Following pharmacists' advice	1 (1)
Taking the benzodiazepine when needed	1 (1)

Reasons for attempted benzodiazepine withdrawal were derived by having participants choose from multiple choice options presented to them with the questions. Strategies used for withdrawing the benzodiazepines were derived by having participants write open‐ended answers in space provided after the questions. The variables presented in the table are thematically collated.

In a binary logistic regression analysis, none of the variables evaluated (age, sex, level of education, benzodiazepines being used, length of action of the benzodiazepine, and reason for taking the benzodiazepine) were significantly associated with a benzodiazepine withdrawal attempt. There was no significant correlation between the BMQ themes and past withdrawal attempts.

### 3.2 *Current* long‐term benzodiazepine use

There was a moderately strong negative correlation (*r*_s_ [75] = −0.390, *p* = 0.001) between current chronic benzodiazepine use and participants' level of education. The correlation between current long‐term use of benzodiazepines in the sample with either of the BMQ‐specific themes was only moderately strong. There was a positive (*r*_s_ [75] = 0.316, *p* = 0.006) correlation of the scores from the BMQ‐specific theme around necessity and a negative (*r*_s_ [75] = −0.338, *p* = 0.003) correlation of the scores from the concerns theme with current long‐term use of benzodiazepines. Details about the correlations between current long‐term benzodiazepine use, level of education, BMQ themes as well as past and future behaviours are presented in Table 3.

**Table 3:** Correlation coefficients and *p* values between current long‐term use, past use behaviour, future use behaviour, BMQ subscales, and sociodemographics

Dependent Variable	Independent Variable	*N*	Correlation Coefficient	*P* Value
***Past benzodiazepine use behaviours (withdrawal attempt)***	Level of education	75	0.141	0.228
	Concerns score from BMQ‐specific	75	0.166	0.155
	Necessity score from BMQ‐specific	75	−0.016	0.891
	***Current*** benzodiazepine use behaviour (use >1 year)	75	−0.076	0.513
	***Future*** benzodiazepine use behaviour	75	0.078	0.506
***Current long‐term benzodiazepine use (use > 1 year)***	Level of education	75	−0.390	0.001
	Concerns score from BMQ‐specific	75	−0.338	0.003
	Necessity score from BMQ‐specific	75	0.316	0.006
	***Future*** benzodiazepine use behaviour	75	−0.236	0.041
***Future benzodiazepine use behaviours (willingness to try behavioural alternatives)***	Level of education	75	0.220	0.058
	Concerns score from BMQ‐specific	75	0.297	0.010
	Necessity score from BMQ‐specific	75	−0.127	0.278

Abbreviation: BMQ‐specific, Beliefs about Medicines Questionnaire‐specific.

We observed no significant correlation between participants' age, sex, benzodiazepines being used, duration of action of the benzodiazepine, or their reason for taking the benzodiazepine with current long‐term benzodiazepine use.

### 3.3 *Future* benzodiazepine use behaviours

In the sample, about two thirds (*n* = 48) of the participants were not willing to consider behavioural therapies as a substitution for their benzodiazepines (only 27/75 participants were interested in behavioural therapies). In these cases, participants' lack of confidence about the efficacy of behavioural therapies and their lack of time to try behavioural therapies were the main two reasons for not considering behavioural therapies. The reported reasons for not considering behavioural therapies are highlighted in Table 4.

**Table 4:** Reported reasons for not preferring behavioural therapies (*n* = 48)

Reasons for Not Preferring Behavioural Therapies	Number (%)
Lack of confidence about behavioural therapies	18 (38)
Lack of time	16 (33)
Dependency on sleeping pill	15 (31)
Participants' perception that behavioural therapies take longer time to produce effect	11 (23)
Participants' perception that seeing a psychologist is costly	9 (19)
Other reason	5 (10)

Reasons for not preferring behavioural therapies were derived by having participants choose from multiple choice options presented to them with the questions.

We observed no statistically significant association between past benzodiazepine use behaviours (withdrawal attempts) and the future intentions of trialling behavioural therapies (Table 3).

There was a weak (*r*_s_ [75] = 0.297, *p* = 0.010) relationship between the BMQ concerns theme and future preference for behavioural therapies (Table 3). Interestingly, binary logistic regression revealed that participants using benzodiazepines for sleep disorders were more likely to be in the “interested in behavioural therapies” group (odds ratio [OR] = 3.138; 95% confidence interval [CI], 1.037‐9.492) (Table 5). Indeed, of the 27 participants who were interested to consider behavioural therapies, 20 participants were those who reported using the benzodiazepine for managing sleep disorders.

**Table 5:** Logistic regression model predicting preference for behavioural therapies based on demographic variables

Factor	OR	95% CI	*P* Value
Age	0.979	(0.947‐1.011)	0.199
Sex	0.711	(0.245‐2.064)	0.530
Using benzodiazepines for sleep	3.138	(1.037‐9.492)	0.043
Using benzodiazepines for at least 1 year	0.496	(0.169‐1.456)	0.202

The dependent variable in this analysis is future preference for behavioural therapies coded as 0 = *not interested in behavioural therapies* and 1 = *interested in behavioural therapies* (target group). Abbreviations: CI, confidence interval; OR: odds ratio.

### 3.4 Source preference for information about alternative behavioural therapies

Doctors were rated as the most preferred source of information for learning more about behavioural therapies (76%, *n* = 57). Following doctors, most of the participants preferred pharmacists as the second (53%, *n* = 40), internet as the third (41%, *n* = 31), and psychologists as the fourth choice (37%, *n* = 28) to consult about behavioural therapies.

## 4 DISCUSSION

To our knowledge, this is the first study exploring the actual beliefs of benzodiazepine users about their medication, their willingness to withdraw or discontinue the benzodiazepine, and future preferences for alternative behavioural therapies. The present study highlights the divergence between Australian benzodiazepine prescribing guidelines and current practice in Australian primary care, where two thirds of our primary care‐based sample participants reported long‐term use (ie, using benzodiazepines for at least 1 year). A few participants had tried different strategies to withdraw from benzodiazepines. Many of these strategies (such as sudden cessation of the benzodiazepine and consuming alcohol and herbal products instead of the medication) were inappropriate. Participants' lower level of education and their beliefs (low concerns and high necessity) appeared to contribute to the current high rate of long‐term benzodiazepine use in this Australian sample. Participants' beliefs appeared not to be associated with their past withdrawal attempts; however, the item used in our questionnaire simply probed whether participants had made any attempt to withdraw their benzodiazepine, not the actual number of attempts made, which may have been a variable associated with the patients concerns about use. We observed, however, an association between concerns about use and future intention to try behavioural alternatives. Using the benzodiazepine for sleep‐related problems was a predictor for willingness to consider alternative behavioural therapies. Only a third of participants expressed an interest in considering behavioural therapies as an alternative to the use of benzodiazepines. Doctors and pharmacists were the two main health professionals of choice with whom benzodiazepine users wanted to discuss potential behavioural therapies. Therefore, it is clear that a collaborative effort in primary care between doctors and pharmacists may help to ensure quality use of benzodiazepines. Based on the study findings, strategies for shifting from ongoing benzodiazepine use to considering behavioural therapies should include counselling patients about the balance between the necessity of using benzodiazepines and the risks associated with ongoing use in the primary care settings.

The observed negative association between the participants' level of education and the current chronic use of benzodiazepines suggests that individuals with a lower level of education perhaps require particular review and targeted information provision. While past withdrawal attempts do indicate previous positive inclination to cease benzodiazepine use, failure at these attempts perhaps reinforces the necessity of continuing the medication as well as reduces the user's perception of their ability to be able to cease use. Therefore, based on theories such as those around planned behaviour^52^, ^53^ as well as our study results, counselling directed at current use and future intention to use is perhaps a more judicious use of clinical time when attempting de‐prescribing of benzodiazepines, rather than focussing on past withdrawal attempts.

The observed positive relationship of the necessity theme and the negative relationship of the concerns theme with actual long‐term benzodiazepine use (*current* behaviour) highlights that the decision to continue benzodiazepines reflected the balance between these two opposing belief sets. However, we observed that future intentions around continued benzodiazepine use (eg, voiced preference for behavioural therapies in the *future*) were only related with the concerns theme, highlighting that if targeted information provision can help build realistic concerns about ongoing use, benzodiazepine users may be swayed to discontinue and switch to behavioural alternatives in the future. The results of this study, therefore, suggest that the BMQ could be a useful instrument to elicit medication beliefs and increase health care professionals' understanding of patients' attitudes towards the medication, which can, in turn, potentially facilitate the de‐prescribing process. Martin et al describe the use of the BMQ‐specific questionnaire to compare patients' beliefs about their benzodiazepines before and after an educational program designed to facilitate decisions about discontinuing benzodiazepines; they report a significantly lower‐necessity score and higher‐concerns score after the intervention.^54^ In our study, the results indicate that concerns about the medication are related to both lower current long‐term use and higher preference for future behavioural alternatives. In fact, health concerns and the fear of being addicted were the most commonly reported reasons for a benzodiazepine withdrawal attempt, as shown in other studies as well.^55^ Therefore, discussing concerns in an individualised manner to the patient may be effective in helping patients decide to discontinue long‐term benzodiazepine use. Since there is considerable evidence around applying patients' beliefs about medication to explore or improve their adherence to a set treatment plan,^56^, ^57^ it can be suggested that patients' beliefs about their benzodiazepine can also be used to predict adherence to a set treatment withdrawal plan.

Sudden cessation of benzodiazepines was the most commonly used strategy for withdrawing the benzodiazepine, and this approach is a distinct departure from clinical guidelines recommending gradual dose reduction for patients on benzodiazepines for longer than 3 to 4 weeks to minimise or avoid withdrawal symptoms.^58^, ^59^ Sudden cessation of benzodiazepines in long‐term users may result in life‐threatening seizures.^58^ These disparities between the guidelines and current withdrawal strategies may reflect that either participant opted to self‐withdraw without health professional advice, or participants possibly received only ad‐hoc advice from their GPs (or primary care physicians or family physicians) or dispensing pharmacists to withdraw the benzodiazepine. In the latter instance, a clear step‐by‐step plan may not have been offered during the GP or pharmacist consultation, given that these health professionals would be well aware of the pharmacological consequences of sudden cessation and would be able to refer to published guidelines on how to help patients discontinue benzodiazepines in a tapered manner.^60^, ^61^, ^62^ Given that even minimal interventions by primary health care professionals can be effective,^63^ primary care professionals should be upskilled and upresourced so as to be able to provide concrete direction when recommending discontinuation of benzodiazepines. A meta‐analysis suggests that gradual dose reduction combined with psychological interventions has better outcomes compared with a gradual dose reduction alone.^64^ Appropriate de‐prescribing plans, adjunctive psychotherapy, and careful monitoring (eg, using withdrawal assessment tools) should be key elements in the benzodiazepine discontinuation process initiated by GPs, who are the main prescribers of benzodiazepines.^58^, ^65^, ^66^ Evidence‐based pragmatic guidelines for de‐prescribing benzodiazepines and practice toolkits may be required to facilitate this.

A majority of the participants were not interested in behavioural therapies, contrary to previous studies generally demonstrating that patients favour non‐pharmacological therapies over pharmacotherapies.^67^, ^68^ This inconsistency relative to other studies may be due to small sample size and selection bias in our study. The inconsistency might also be related to the fact that the participants may not have been experienced or informed about behavioural treatments earlier.^67^, ^69^ Greater public awareness about the efficacy of behavioural therapies may be a prudent step for addressing this issue, eg, through public health campaigns given that benzodiazepines have been implicated in vehicle accidents.^70^, ^71^ Another strategy to improve the uptake of behavioural therapies could be to introduce internet‐delivered behavioural therapy services. Studies suggest that internet‐based behavioural therapies have the potential to be time‐efficient and cost‐effective as well as quite acceptable for patients.^72^, ^73^ Interestingly, participants with sleep problems in this study were more likely to prefer behavioural treatment, which supports and further extends previous research.^74^, ^75^ These patients can be specifically targeted for behavioural intervention in primary care settings.

Since a majority of the participants identified doctors as their first choice for learning more about behavioural therapies, GPs need to take the initiative to make patients familiar with the behavioural therapies. However, physicians cite time paucity, high workload, lack of skills in behavioural treatment, and limited accessibility to behavioural therapy providers as the barriers to reduce benzodiazepine use and introduce behavioural interventions in general practice setting.^76^, ^77^ Introducing other health care professionals co‐located within GP practice centres to support GPs for educating patients about behavioural therapies may help to overcome some of these issues.^29^ In a qualitative Australian study, GPs acknowledged the role of other health professionals in facilitating successful benzodiazepine cessation.^44^ In the current study, following GPs, pharmacists were the second preferred source of information for behavioural therapies. Thus, a collaborative approach with pharmacists could be an option where they can support GPs in withdrawing benzodiazepines and providing behavioural therapies.^78^ Community pharmacists also have the opportunity to counsel patients at the point of purchase; in the case of patients with repeat prescriptions, community pharmacists see benzodiazepine users even more frequently. At these opportunities, pharmacists can discuss the potential risks of long‐term ongoing use, provide information about alternatives, and refer patients to their doctor for a withdrawal or discontinuation plan. Once patients have a plan, in an interdisciplinary model, pharmacists should be apprised of the plan (which could be written out as a prescription) for further facilitating patient adherence to the plan with supportive advice. Indeed, data suggest that community pharmacists do frequently encounter chronic benzodiazepine use in practice, but fewer than half converse with the patient about ongoing use.^79^, ^80^ On the other hand, a recent study in Australia highlighted that pharmacists could be successfully trained to deliver behavioural interventions with positive sleep health outcomes in patients with insomnia,^81^ suggesting the benefit of developing pharmacist roles in this area. Despite the potential to play key roles in benzodiazepine deprescribing,^82^, ^83^ pharmacists, especially community pharmacists, are currently underutilised.^79^

### 4.1 Strengths and limitations

The small sample size may be a limitation of this study. For moderate strength correlations (eg, 0.30‐0.40), a sample of 75 may be sufficient to demonstrate significance at a 0.05 significance level with 75% power.^84^ However, the study may have been underpowered to demonstrate lower‐strength correlation at the same significance level and power. There is a possibility of selection bias, as individuals who have successfully withdrawn their benzodiazepines were not recruited for this study. Further, the questions used in this study to explore participants' willingness to withdraw and preferences for behavioural therapies were customised for this study. However, the questionnaire was developed by reviewing the previous literature and was face‐validated by psychology, sleep, and pharmacy practice researchers. Given this part of the questionnaire measured a set of diverse issues (past behaviour of withdrawing, actual daily use of benzodiazepine, and future intentions to try alternatives to benzodiazepine use), internal consistency was not measured, as behaviour and intention constructs are in themselves quite different and these questions also had different response items. Past behaviours appeared less important based on our data, and current behaviours are verifiable through pharmacy prescription records. Therefore, it may be suggested that a fuller set of items with comparable response sets around intended future behaviours with respect to benzodiazepines should be constructed and tested psychometrically. This would be useful for future research and is certainly a limitation in our study. The questionnaire did not ask explicitly about participants' readiness or current intention to withdraw from the benzodiazepine. Exploring participants' belief was limited to BMQ‐specific rather than using the more comprehensive exploration of belief sets, for example, using variables included in models such as the HBM. Lastly, there is a possibility for response bias (eg, strong beliefs about the necessity of benzodiazepines, low concerns, and social desirability).

## 5 CONCLUSION

Specific characteristics of benzodiazepine users and their beliefs about taking the benzodiazepine can inform the provision of individualised interventions by GPs to help switch patients currently on long‐term benzodiazepines to alternative behavioural therapies. This study highlights the significance of informing patients about the balance between the necessity of use versus the concerns that long‐term use of benzodiazepines raises. Given that GPs are very time pressured, introducing practice pharmacists within general practices could be time efficient and enhance GPs' capacity for providing behavioural therapies, and this area of collaborative care in de‐prescribing unwarranted use of high‐risk medications warrants future research.

## FUNDING INFORMATION

This research did not receive any specific grant from funding agencies in the public commercial or not‐for‐profit sectors. However, the authors acknowledge the PhD scholarship support received by the first author from NeuroSleep, a Centre of Research Excellence supported by a grant from the Australian National Health and Medical Research Council. The funding sources were not involved in study design; collection, analysis, or interpretation of data; writing of the report; or the decision to submit the report for publication.

## CONFLICTS OF INTEREST

None.

## AUTHOR CONTRIBUTIONS

Conceptualization: Fatema‐Tun‐Naher Sake, Keith Wong, Bandana Saini

Formal Analysis: Fatema‐Tun‐Naher Sake, Keith Wong, Bandana Saini

Writing (original draft): Fatema‐Tun‐Naher Sake

Writing (review and editing): Fatema‐Tun‐Naher Sake, Keith Wong, Delwyn Bartlett, Bandana Saini

All authors have read and approved the final version of the manuscript.

Fatema‐Tun‐Naher Sake had full access to all of the data in this study and takes complete responsibility for the integrity of the data and the accuracy of the data analysis.
